# Functional analysis of the BRI1 receptor kinase by Thr-for-Ser substitution in a regulatory autophosphorylation site

**DOI:** 10.3389/fpls.2015.00562

**Published:** 2015-07-30

**Authors:** Man-Ho Oh, Kyle W. Bender, Sang Y. Kim, Xia Wu, Seulki Lee, Ill-Sup Nou, Raymond E. Zielinski, Steven D. Clouse, Steven C. Huber

**Affiliations:** ^1^Plant Developmental Genetics, Department of Biological Science, College of Biological Sciences and Biotechnology, Chungnam National UniversityDaejeon, South Korea; ^2^Protein Biochemistry, Department of Plant Biology, University of IllinoisUrbana, IL, USA; ^3^U.S. Department of Agriculture, Agricultural Research ServiceUrbana, IL, USA; ^4^Department of Genome Sciences, University of WashingtonSeattle, WA, USA; ^5^Department of Horticulture, Sunchon National UniversitySunchon, South Korea; ^6^Department of Horticultural Science, NC State UniversityRaleigh, NC, USA

**Keywords:** BRI1, kinase domain, directed mutagenesis, autophosphorylation

## Abstract

BRI1 becomes highly phosphorylated *in vivo* upon perception of the ligand, brassinolide, as a result of autophosphorylation and transphosphorylation by its co-receptor kinase, BAK1. Important autophosphorylation sites include those involved in activation of kinase activity and those that are inhibitory, such as Ser-891. The inhibitory sites are autophosphorylated after kinase activation has been achieved and are postulated to contribute to deactivation of the kinase. The function of phosphosites is usually tested by substituting a non-phosphorylatable residue or an acidic residue that can act as a phosphomimetic. What has typically not been examined is substitution of a Thr for a Ser phosphosite (or vice versa) but given that Thr and Ser are not equivalent amino acids this type of substitution may represent a new approach to engineer regulatory phosphorylation. In the present study with BRI1, we substituted Thr at the Ser-891 phosphosite to generate the S891T directed mutant. The recombinant Flag-BRI1 (S891T) cytoplasmic domain protein (the S891T protein) was catalytically active and phosphorylation occurred at the engineered Thr-891 site. However, the S891T recombinant protein autophosphorylated more slowly than the wild-type protein during expression in *E. coli*. As a result, activation of peptide kinase activity (measured *in vitro*) was delayed as was transphosphorylation of bacterial proteins *in situ*. Stable transgenic expression of BRI1 (S891T)-Flag in Arabidopsis *bri1-5* plants did not fully rescue the brassinosteroid (BR) phenotype indicating that BR signaling was constrained. Our working model is that restricted signaling in the S891T plants occurs as a result of the reduced rate of activation of the mutant BRI1 kinase by autophosphorylation. These results provide the platform for future studies to critically test this new model *in vivo* and establish Ser-Thr substitutions at phosphosites as an interesting approach to consider with other protein kinases.

## Introduction

Plants contain a much expanded receptor-like kinase family of proteins with more than 600 members in Arabidopsis (Shiu and Bleecker, 2001). Two of the most studied receptor kinases in plants include BRASSINOSTEROID INSENSITIVE 1 (BRI1) and BRI1-ASSOCIATED RECEPTOR KINASE 1 (BAK1), which function together as receptor and co-receptor, respectively, in brassinosteroid (BR) signaling (Vert et al., 2005; Clouse, 2011). The BR ligand, brassinolide (BL), binds to the extracellular domain of BRI1 (Kinoshita et al., 2005; Sun et al., 2013), and serves as the “molecular glue” to stabilize the association of BAK1 as co-receptor (Santiago et al., 2013). However, it is not entirely clear whether BL binds to preformed BRI1 homodimers, which then facilitates BAK1 binding, or to a small pool of preformed BRI1-BAK1 heterooligomers (Bücherl et al., 2013). Regardless of which scenario is correct, the notion is that BL binding to BRI1 results in some basal activation allowing transphosphorylation and release of the BRI1 KINASE INHIBITOR1 (BKI1) (Jaillais et al., 2011; Wang et al., 2014), which then allows further activation of BRI1 and BAK1 via autophosphorylation and sequential transphosphorylation of both receptor kinases (Wang et al., 2005a,b, 2008). An alternative view (Wang et al., 2014) is that when the cytoplasmic domains of BRI1 and BAK1 are juxtaposed in the presence of ligand, it is the basal activity of BAK1 that initially transphosphorylates and activates BRI1, allowing for phosphorylation and release of the BKI1 inhibitor protein. This scenario is also consistent with the observation that Somatic Embryogenesis Receptor Kinases (SERKs) are absolutely required for the early events of brassinosteroid signaling (Gou et al., 2012). In either case, the activated BRI1-BAK1 signaling complex then initiates further downstream BR signaling ultimately resulting in the regulation of gene expression (Vert et al., 2005; Vert and Chory, 2006; Wang et al., 2012).

Much remains to be learned about the initial phosphorylation events involved in BR signaling and the functional impact of site-specific phosphorylation of the cytoplasmic domains of the BRI1 and BAK1 receptor kinases. The activation of kinase activity almost certainly involves phosphorylation of residues within the activation loops (Oh et al., 2000; Wang et al., 2008) of these arginine-aspartate (RD)-type protein kinases (Johnson et al., 1996). In addition, residues outside of the activation loop can also be stimulatory to kinase activity. For example, the carboxy (C)-terminus of BRI1 is inhibitory to kinase activity and phosphorylation of residues within the C-terminus, such as Thr-1180 and Ser-1162, relieve the inhibition (Wang et al., 2005a). Furthermore, phosphorylation of residues can be essential for receptor kinase function *in vivo* even though they have no impact on kinase activity *in vitro*. An example of this category would be Tyr-610 of BAK1; phosphorylation of Tyr-610 is essential for BR signaling *in vivo* but not for BAK1 kinase domain activity (Oh et al., 2010). Conversely, phosphorylation of some sites can also inhibit receptor kinase activity. Examples of inhibitory autophosphorylation sites include BRI1 Tyr-831 and Tyr-956 (Oh et al., 2009) and Ser-891 (Oh et al., 2012c). Phosphorylation of these sites potentially contributes to deactivation of BRI1 and termination of signaling.

The current study focuses on manipulation of the putative Ser-891 phosphosite. Evidence consistent with phosphorylation of BRI1 at the Ser-891 site has come from several lines of study. It was originally identified as an ambiguous site by a MALDI-mass spectrometry based study of *in vitro* phosphorylated BRI1 cytoplasmic domain protein that identified a single phosphorylation site in the 10 amino acid peptide DS^887^LIGS^891^GGFG (Oh et al., 2000). Subsequent investigation of *in vivo* phosphorylation sites by LC-MS/MS failed to detect phosphorylation of either Ser-887 or Ser-891, but did unambiguously identify Thr-872 and Thr-880 as true *in vivo* phosphorylation sites within the tryptic phosphopeptide L**pT**^872^FAD LLQA**pT**^880^ NGFHNDS^887^ LIGS^891^GGF GDVYK (where pT in bold is phosphothreonine) derived from immunopurified BRI1-Flag (Wang et al., 2005b). To further study the possible phosphorylation of Ser-891, we developed a custom-made pSer891-specific antibody that reacted with recombinant BRI1-Flag cytoplasmic domain protein but not the S891A directed mutant, which retained full kinase activity as evidenced by retention of autophosphorylation on other sites as well as peptide kinase activity *in vitro* (Oh et al., 2012c). Thus, the anti-pS891 antibodies appeared to be sequence- and modification-specific. Importantly, the anti-pS891 antibodies reacted with BRI1-Flag protein affinity purified from Arabidopsis seedlings suggesting that phosphorylation at this site occurs *in vivo*, although there is still no mass spectrometry based evidence to support this site identification. However, studies with the anti-pS891 antibodies indicated that phosphorylation at this site occurred slowly and increased with time (up to 12 h) following addition of brassinolide to liquid-culture grown Arabidopsis seedlings, whereas other sites were phosphorylated more rapidly. Previous mass spectrometry-based studies of BRI1 phosphorylation have used seedlings harvested after only 1.5 h of brassinolide treatment (Wang et al., 2005b), where the site stoichiometry is not maximum. As well, lack of identification of a phosphosite by mass spectrometry is a negative result that does not mean that the phosphosite does not exist. Moreover, substitution of Asp at the Ser-891 site as a phosphomimetic resulted in recombinant protein with reduced autophosphorylation activity *in situ* as well as reduced peptide kinase activity *in vitro* (43% of wild type protein) and transgenic plants expressing BRI1 (S891D)-Flag in the bri1-5 background were severely dwarfed and had several characteristics consistent with impaired BR signaling (Oh et al., 2012c). The focus of the current study was to determine the impact of a Ser-for-Thr substitution at the position 891.

The functional role of phosphorylation can be studied one of two ways: (1) correlation of phosphorylation stoichiometry with changes in kinase activity; and (2) site-directed mutagenesis of the phosphosite. The latter is the more common and most direct approach and involves substitution of the Ser, Thr, or Tyr residue with a conservative but non-phosphorylatable residue (e.g., Ala or Gly for Ser or Thr; and Phe for Tyr). However, if this substitution inhibits kinase activity it is not clear whether the −OH group is essential for structure/activity or whether the phosphorylated residue is essential for activity. Conversely, substitution of an acidic residue (Asp or Glu) for the phosphorylated residue may serve as a phosphomimetic, especially if introduction of negative charge at that position is the primary functional effect of phosphorylation. However, loss of activity is difficult to interpret unequivocally.

In addition to helping define the functional role of site-specific phosphorylation, directed mutagenesis can also be an effective approach to engineer receptor kinase activity. For example, phosphorylation of BRI1 Tyr-831 appears to attenuate BRI1 kinase activity *in planta* because transgenic Arabidopsis plants expressing BRI1 (Y831F)-Flag have enhanced BR signaling and increased growth (Oh et al., 2011). Similarly, using a combination of immunological and transgenic approaches, we previously identified Ser-891 as an inhibitory autophosphorylation site of BRI1 kinase activity and BR signaling (Oh et al., 2012c). Serine-891 is located in the ATP-binding domain (G-loop) and inhibition of kinase activity by phosphorylation of this residue is not surprising. In the original study (Oh et al., 2012c), we found that many different residues including hydrophobic and basic amino acids could be substituted for Ser-891 without appreciable inhibition of BRI1 kinase activity. However, substitution of Thr for Ser-891 was not tested. Threonine for serine (or vice versa) substitution represents another opportunity for engineering receptor kinase function since phosphorylation of Ser vs. Thr appears to be unequal in terms structural effects on the protein in question (Elbaum and Zondlo, 2014). In the present study, we characterized the S891T mutant expressed as the recombinant cytoplasmic domain Flag-BRI1 (S891T) protein produced in *E. coli*, and in the full length BRI1 (S891T)-Flag protein expressed in transgenic Arabidopsis plants and uncovered distinct regulatory properties of the S891T mutant that are linked to altered BR signaling in plants. The results suggest that Ser and Thr residues are not always equivalent and can have different effects as phosphosite residues.

## Materials and methods

### Plant growth and transformation

*Arabidopsis thaliana* ecotype Ws-2 was used as the wild-type and mBRI1-Flag, BRI1-Flag and BRI1 (S891T)-Flag transgenic lines in the *bri1-5* mutant background were produced as previously described Oh et al. (2009). The BRI1–Flag construct was the template for site-directed mutagenesis with the QuikChange XL Site-Directed Mutagenesis Kit (Stratagene) to generate the site-directed mutant, S891T, which was transformed into the *bri1-5* mutant as previously described Wang et al. (2005b) using the floral dip method (Clough and Bent, 1998). All constructs were sequenced in both directions to verify specific mutations and lack of additional mutations.

### Preparation of microsomal membranes and immunoblot analysis of proteins

*A. thaliana* plants were grown in shaking liquid culture and microsomal membranes were isolated as described Wang et al. (2005b). Membranes were solubilized with 1% (vol/vol) Triton X-100, clarified by centrifugation, and diluted so that protein concentration was adjusted to 0.5–1.0 mg/mL, and Triton X-100 was reduced to 0.1%. BRI1-Flag was then immunoprecipitated with prewashed anti-Flag M_2_ affinity gel (Sigma–Aldrich). Immunoprecipitated full-length BRI1-Flag proteins were subjected to SDS-PAGE followed by transfer to PVDF membranes and immunoblot analysis performed using anti-Flag antibodies (1:5000 dilution), anti-phosphothreonine antibodies (1:500 dilution; Invitrogen, Carlsbad, CA, USA) and anti-phosphotyrosine antibodies (1:500 dilution). Several of the custom antibodies used were described previously (Oh et al., 2012c) and are listed here with the corresponding phosphopeptide antigens: pS891, DSLIGpS^891^GGFGD; pS858, KEALpS^858^INLAA; and pT872, PLRKLpT^872^FADL). The anti-pS963 antibodies were generated against the phosphopeptide antigen: KYGpS^963^LEDVLHDPKK. All custom antibodies were produced by GenScript and sequentially affinity purified using the nonphosphorylated and then the phospho-containing antigen peptides. Immunoblots involving fluorescent secondary antibodies (IRDye 800CW; LI-COR Biosciences, Lincoln, NE, USA) were scanned using an Odyssey Infrared Imaging System (LI-COR Bioscience) for visualization, while immunoblots developed using chemiluminescent detection were imaged using a C-DiGit Blot Scanner (LI-COR Biosciences).

### Recombinant protein analysis and *in vitro* peptide kinase assay

The Flag-BRI1 cytoplasmic domain construct (Oh et al., 2000) was the template for site-directed mutagenesis with the QuikChange XL Site-Directed Mutagenesis Kit (Stratagene, La Jolla, CA, USA) to generate the site-directed mutants, BRI1(S891T)-Flag, BRI1(T880A)-Flag, and the double mutants T880A/S891A and T880A/S891T, for expression as a recombinant proteins in *E. coli*. After sequencing to confirm mutated regions, vectors containing genes of interest were introduced to *E. coli* BL21 (DE3) cells (Novagen, Gibbstown, NJ, USA) through plasmid transformation. Cultures were induced with 0.3 mM IPTG at 23°C for variable periods of time up to16 h and the soluble recombinant protein produced was purified using anti-Flag M2 affinity gel (Sigma-Aldrich, St. Louis, MO, USA). After elution from the beads, the protein solution was dialyzed against a 1000 × volume of buffer containing 20 mM Mops, pH 7.5, and 1 mM DTT. *E. coli* cells were grown in LB media or TB media as specified. Peptide substrate phosphorylation assays were performed as described (Oh et al., 2000) using the BR13 peptide (sequence: GRJKKIASVEJJK, where J is norleucine; produced by Bethyl Laboratories, Montgomery, TX, USA).

### Transcript analysis

Total RNA was extracted from the 7 day old seedlings grown on long day condition (16 h light/8 h dark) using Qiagen RNeasy mini kit. For RT-PCR, 26 and 30 cycles were used for UBQ and BRI1, respectively. The primer sequence for the genes are following. UBQ-Forward: GATCTTTGCCGGAAAACAATTGGAGGATGGT; UBQ-Reverse: CGACTTGTCATTAGAAAGAAAGAGATAACAGG; BRI1-Forward: TCCGCGGTGTGATCCTTCAAAT; BRI1-Reverse: GCCGTGTGGACCAGTTTA GTTT.

## Results and discussion

### Autophosphorylation of Flag-BRI1 (S891T) at the Thr-891 site

**Figure 1:**
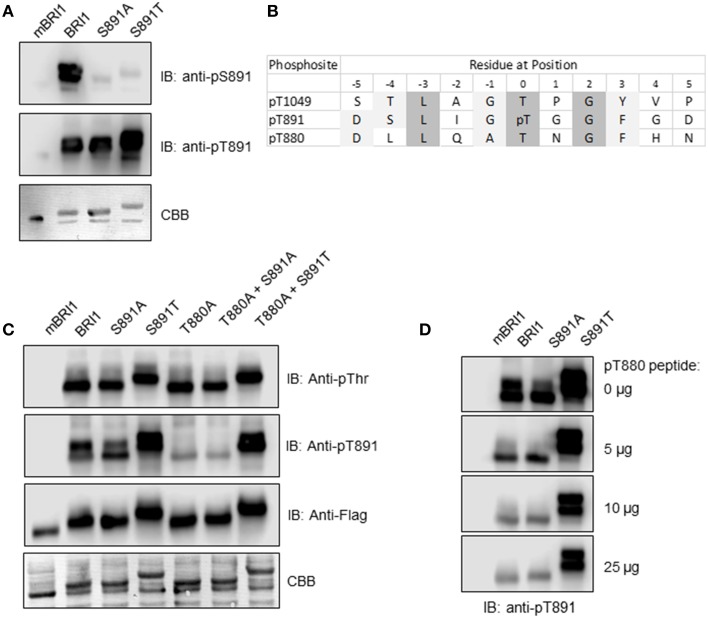
**Phosphorylation of the recombinant Flag-BRI1 (S891T) mutant at the engineered Thr-891 site. (A)** Immunoblot analysis of recombinant BRI1 proteins autophosphorylated during production in *E. coli*, using previously described anti-pS891 antibodies and anti-pT891 antibodies developed in this study. **(B)** Sequence alignment of BRI1 showing two known autophosphorylation sites with significant similarity to the antigen sequence used to generate the anti-pT891 antibodies. Light and dark shading represent similar and identical amino acids, respectively. **(C)** Reaction of various antibodies with mBRI1, BRI1, and selected directed mutants. **(D)** Peptide competition of the reaction of anti-pT891 antibodies with BRI1 and the S891A and S891T mutants. Increasing amounts of the pT880 synthetic peptide (sequence: DLLQApTNGNF, where pT is phosphothreonine) were added to the primary antibodies as indicated.

In previous studies, immunoblotting with custom antibodies (anti-pS891 antibodies) provided one line of evidence that autophosphorylation occurred at the Ser-891 site that is located within the Gly-rich loop (G-loop) of subdomain I (GS^891^GGFG). The G-loop is involved in binding of substrate ATP (Oh et al., 2012c) and as expected, phosphorylation of Ser-891 inhibited BRI1 peptide kinase activity *in vitro* and the phosphomimetic S891D mutant had low activity. However, a variety of other amino acids could be substituted for Ser-891 without loss of autophosphorylation activity including hydrophobic (Phe), basic (Lys, Arg), polar (Gln), and neutral, non-polar (Ala, Gly) residues. Therefore, we anticipated that Thr would readily substitute for Ser. To establish autophosphorylation at the Thr-891 site in the directed mutant, we produced custom antibodies (referred to as anti-pT891 antibodies) against the sequence DSLIGpT^891^GGFD. The same sequence, but with phosphoserine replacing phosphothreonine (pT), was used previously to produce the anti-pS891 antibodies (Oh et al., 2012c). These two antibodies were used to probe recombinant BRI1, the kinase inactive mBRI1, and the S891A and S891T directed mutants. As shown in Figure 1A, recombinant Flag-BRI1 CD reacted with the anti-pS891 antibodies whereas mBRI1 and the directed mutants did not and thus are phosphoserine- and site-specific. This result confirms previous work (Oh et al., 2012c) identifying Ser-891 as an autophosphorylation site using immunological methods. The anti-pT891 antibodies reacted most strongly with the S891T mutant but there was also some reaction with BRI1 and the S891A mutant. Thus, the anti-pT891 antibodies appeared to be phosphospecific (as there was no reaction with mBRI1) but not strictly site-specific. Comparison of the antigen sequence with the BRI1 cytoplasmic domain sequence identified the sequences surrounding Thr-880 and Thr-1049 as having significant similarity that could account for the lack of site specificity. Both Thr-880 and Thr-1049 are recognized to be autophosphorylation sites and are aligned with the pT891 residue in Figure 1B. Thr-1049 is located in the BRI1 activation loop, which contains several phosphorylated residues when activated including not only Thr-1049 but also Ser-1044 and Thr-1045 (Wang et al., 2005b, 2008); such multisite phosphorylation might restrict reaction with the anti-pT891 antibodies.

One approach to identify other phosphosites that might be recognized by the anti-pT891 antibodies would be to test for antibody reaction with BRI1 mutants that had Ala substitutions for each of the candidate phosphosites. Unfortunately, BRI1 (T1049A) is kinase inactive (Wang et al., 2005b, 2008), which precludes this approach for the Thr-1049 site. We previously showed that BRI1 (T880A) retained autophosphorylation activity similar to wild tpe BRI1 and was able to rescue the *bri1-5* mutant phenotype (Wang et al., 2005b). Here, we demonstrate that the recombinant Flag-BRI1 (T880A) protein autophosphorylated strongly on Thr residues during production in *E. coli* (Figure 1C). The T880A mutant protein reacted only weakly with the anti-pT891 antibodies, which we postulate reflects some recognition of the pT1049 sequence as discussed above. The low level of reaction was also observed with the T880A/S891A double mutant, confirming that the anti-pT891 antibodies do not cross react with the pS891 sequence (i.e., the antibodies are phosphothreonine specific). In analogous fashion, BRI1 and the S891A mutant reacted to a similar extent with the anti-pT891 antibodies, which we interpret as recognition of the phosphothreonine-880 (pT880) sequence and some weak recognition of pT1049. With the S891T mutant, all three putative sites would be present and could react with the anti-pT891 antibodies, whereas with the T880A/S891T mutant, only the pT891 site and weakly reactive pT1049 site would be present. Collectively, these results suggest that phosphorylation of the Thr-891 site occurs in the S891T mutant but that immunoblot signals also reflect some reaction with the pT880 site and a minor reaction with the pT1049 site.

It is likely that the polyclonal anti-pT891 antibodies consist of a mixture of antibodies that recognize specific features of the pT891 antigen sequence. If so, it may be possible to selectively block the recognition of the pT880 site by pretreating the anti-pT891 antibodies with the pT880 phosphopeptide (sequence: DLLQATpTNGF). This was indeed observed (see Figure 1D). Adding increasing amounts of the pT880 phosphopeptide reduced the reaction of the anti-pT891 antibodies with BRI1 and the S891A mutant to a low level similar to the weak immunoblot signal attributed earlier to some recognition of the pT1049 site. In contrast, reaction with the S891T mutant protein was reduced but remained high and constant reflecting recognition of pT891 (and a small amount of pT1049). The main conclusion to draw from these experiments is that phosphorylation does occur at the Thr-981 site in the S891T mutant protein. This would perhaps be expected but is not a trivial demonstration because Ser and Thr residues are not always equivalent amino acids (Ubersax and Ferrell, 2007; Elbaum and Zondlo, 2014).

### Activation of Flag-BRI1 (S891T) cytoplasmic domain in *E. coli*

**Figure 2:**
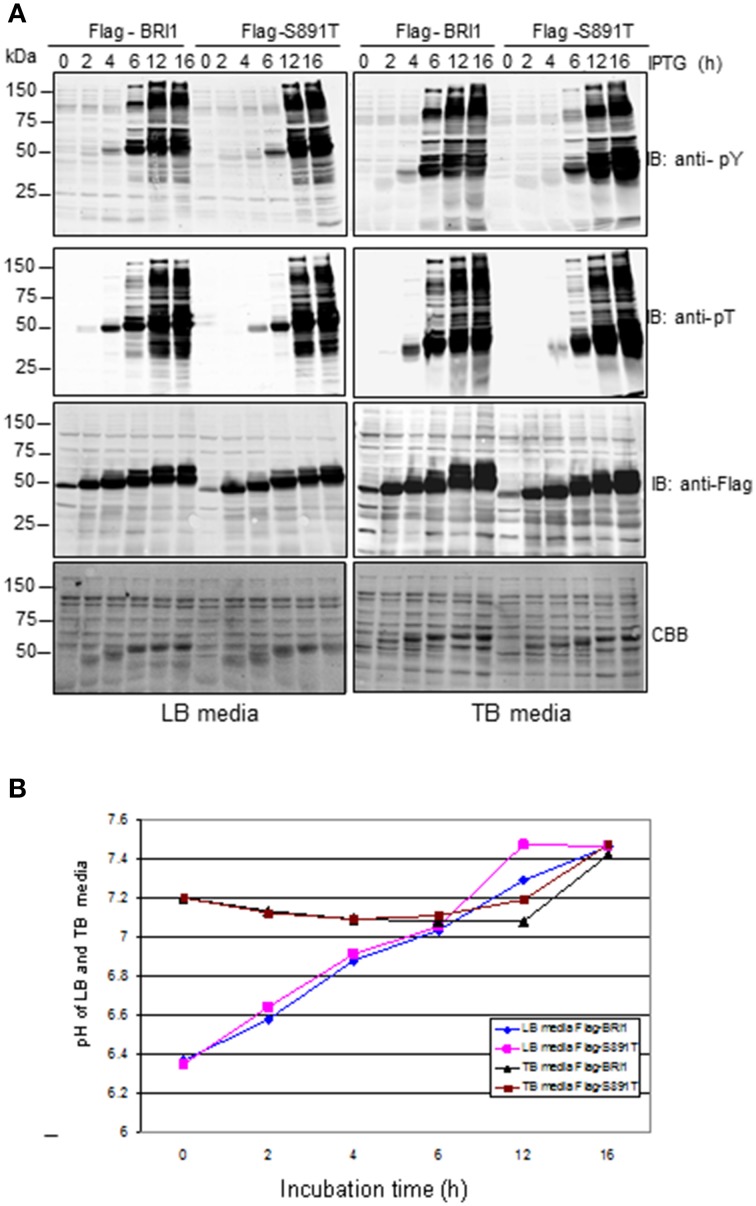
**Autophosphorylation of BRI1 and transphosphorylation of bacterial proteins during expression of Flag-BRI1 and Flag-BRI1 (S891T) in LB or TB media**. **(A)** Immunoblot analysis of soluble cell lysates using generic anti-phosphotyrosine (anti-pY) or anti-phosphothreonine (anti-pT) antibodies. **(B)** Changes in medium pH during induction of BRI1 proteins in LB or TB media.

How the S891T substitution impacts BRI1 kinase activity is of interest and can potentially be monitored several different ways. One simple system to monitor the activity and specificity of a protein kinase is to assess the time course of autophosphorylation of the kinase and transphosphorylation of bacterial proteins during production of the recombinant protein kinase (Wu et al., 2012). In the experiment presented in Figure 2, total cell lysates were analyzed in order to provide an overview of the recombinant BRI1 protein being produced and all soluble *E. coli* proteins. We compared the activation kinetics of wild-type Flag-BRI1 with the S891T mutant, expressed in *E. coli* cells cultured in LB media as in previous experiments. The CBB-stained panel show that at 2 h of induction, the recombinant wild type Flag-BRI1 and S891T mutant protein bands were apparent and increased in abundance with time as did the apparent molecular mass of the proteins. The increase in apparent molecular mass reflects to a large extent the autophosphorylation of the protein at numerous Ser, Thr, and Tyr sites, because when analyzed on a 2-dimensional gel, the BRI1 protein migrates as a series of spots along a diagonal line (Oh et al., 2012a). The most important point to note from Figure 2A (left panel), is that when expressed in LB media, the wild-type BRI1 protein activated by autophosphorylation slightly earlier than the S891T mutant and also initiated transphosphorylation of bacterial proteins at an earlier stage of induction.

In these and previous studies (Oh et al., 2012b; Wu et al., 2012), *E. coli* cultures were grown in LB media, which contains no buffer and is known to change pH with time. Although *E. coli* is a neutrophilic bacterium and grows optimally between pH 6 to 8, primary proton pumps operate over this range to maintain a rather constant internal pH of approximately 7.6 (Padan et al., 1981). Therefore, it was of interest to compare the time course of BRI1 protein production and autophosphorylation in cell cultured in unbuffered LB media with TB media, which is buffered with phosphate to minimize changes in medium pH. Indeed, over the course of our induction experiments, external pH increased by nearly 1 pH unit in LB media, whereas it was relatively constant in TB media (Figure 2B). Even though internal pH is rather constant when external pH changes (Padan et al., 1981), changes in proton pump activity and membrane potential could impact bacterial cell metabolism and the time course of recombinant protein production. Consequently, we expressed wild-type BRI1 and the S891T mutant in TB media as well (Figure 2A, right panels). In general, the results were very similar to those obtained in LB media with Flag-BRI1 activating phosphorylation events earlier compared to the S891T mutant. Because the time courses were very similar between the two culture media, the changes in external pH that occur in LB media seems to not play an important role.

**Figure 3:**
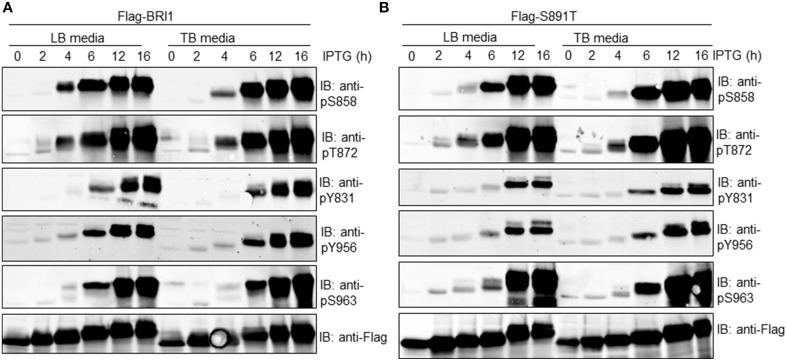
**Immunoblotting analysis of affinity-purified (A) Flag-BRI1 or (B) Flag-BRI1(S891T) protein harvested from cells at different stages of induction using modification-specific antibodies**. Blots were probed with the indicated site- and modification-specific antibodies. Immunoblotting with anti-Flag antibodies demonstrated equal protein loading (1.5 μg per lane) and the electrophoretic mobility shift accompanying autophosphorylation.

In order to more clearly discern the time course of autophosphorylation at specific phosphosites, the Flag-BRI1 and S891T mutant proteins were affinity purified at different times of induction and analyzed by immunoblotting. As shown in Figure 3 (left panel), Flag-BRI1 autophosphorylation increased with time of induction but the kinetics of phosphorylation on specific sites varied with the site. For example, autophosphorylation at the Ser-858 and Thr-872 sites was first evident at 4 h, whereas phosphorylation at the Tyr-831, Tyr-956, and Ser-963 sites was first apparent after 6 h of induction. Phosphorylation at all sites increased with time of induction indicating increased stoichiometry at the sites. Autophosphorylation of recombinant Flag-BRI1 cytoplasmic domain protein at the Ser-963 site was unambiguously identified recently by LC-MS/MS (Wu et al., 2012) and is confirmed in the present study using phosphosite-specific antibodies.

In general, similar patterns for site-specific autophosphorylation were observed when cells were cultured in LB or TB media, indicating that changes in culture pH (Figure 2B) and/or its impact on bacterial cell metabolism, was not playing a role in the overall time course of autophosphorylation at the specific sites monitored. In general, very similar patterns were observed for autophosphorylation of the S891T mutant (Figure 3, right panel), except that autophosphorylation of most sites was slightly delayed compared to the wild type Flag-BRI1. For example, phosphorylation at the Ser-858 site was much weaker at 4 h (compared to Flag-BRI1) but then increased dramatically at later time points. The one exception was phosphorylation at the Thr-872 site, which followed very similar kinetics to that observed for Flag-BRI1. Collectively, the results are consistent with our earlier findings (Oh et al., 2012a) that BRI1 autophosphorylation in *E. coli* is post-translational rather than co-translational, and is hierarchical in nature (some sites appear before others). The novel point added here is that the apparent hierarchical nature is not related to changes in external pH (or its impact on bacterial cell metabolism) based on the similar results obtained in LB vs TB media. Second, the S891T mutant appeared to autophosphorylate more slowly than the wild-type Flag-BRI1. The exception was phosphorylation of the Thr-872 site, which may function to inhibit BRI1 kinase activity (Wang et al., 2005b) and conceivably could contribute to the delayed autophosphorylation/activation observed.

**Figure 4:**
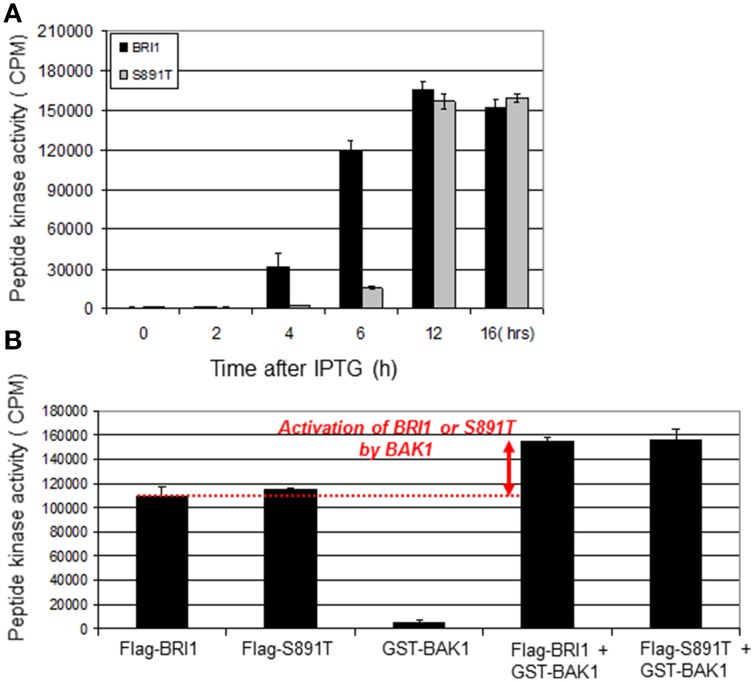
**Peptide kinase activity of Flag-BRI and Flag-BRI1 (S891T). (A)** Peptide kinase activity of Flag-BRI1 and the S891T directed mutant purified at different stages of induction. The BR13 peptide (sequence: GRJKKIASVEJJKK, where J is norleucine) was used in reactions run for 20 min at room temperature. **(B)** Activation of the peptide kinase activity of Flag-BRI1 or the S891T mutant by GST-BAK1. The BR13 synthetic peptide was used as substrate. The increase in BRI1 activity requires an active BAK1 kinase, which alone does not readily phosphorylate the BR13 peptide. Values are means ± SEM; *n* = 3.

BRI1 will readily phosphorylate the BR13 synthetic peptide (Oh et al., 2000), which provides a measure of the activation state of the recombinant receptor kinase. Therefore, it was of interest to monitor peptide kinase activity of Flag-BRI1 and the S891T mutant protein purified at different times after IPTG induction. As shown in Figure 4A and consistent with earlier findings (Oh et al., 2012a), peptide kinase activity of Flag-BRI1 protein was first observed at 4 h of induction and increased to reach a maximum at 12 h of induction, after which there was a slight decrease. The increase in activity reflects autophosphorylation of residues that are essential (e.g., activation loop residues) or stimulatory (e.g., residues in the C-terminal domain) for activity. The decrease in activity from 12 to 16 h, which was relatively small with the time points analyzed in the present study, can occur as a result of autophosphorylation of residue(s) that inhibit kinase activity, such as Ser-891 (Oh et al., 2012c). The most important point to note is that activation of the S891T mutant in *E. coli* was delayed relative to Flag-BRI1, but at 12-h of induction had achieved a similar activity level. The delay in activation of peptide kinase activity is consistent with the delay in autophosphorylation of most sites (Figure 3) and delayed transphosphorylation of bacterial proteins *in situ* (Figure 2A).

Another relevant assay that involves the peptide kinase activity of BRI1 and reflects the functional interaction between BRI1 and BAK1 involves BAK1 transactivation of BRI1 *in vitro*; this functional interaction is thought to reflect their interaction *in vivo* and to be essential for robust BR signaling in planta (Wang et al., 2008). The result of the transphosphorylation is enhanced BRI1 kinase activity that can be measured *in vitro* with the peptide kinase assay (Wang et al., 2008; Oh et al., 2014). As shown in Figure 4B, and as expected, both Flag-BRI1 and the S891T mutant readily phosphorylated the BR13 synthetic peptide whereas GST- BAK1 did not. Moreover, GST-BAK1 substantially increased the ability of both Flag-BRI1 and the S891T mutant to transphosphorylate the synthetic peptide substrate indicating that once activated, the S891T mutant interacted with BAK1 similar to the wild type Flag-BRI1 protein. The important conclusion is that BAK1 could activate the S891T mutant in an equivalent manner to wild-type BRI1, which is recognized to be an important factor that regulates signaling leading to plant growth (Vert et al., 2005; Clouse, 2011).

### Structural modeling of the S891T mutant protein

**Figure 5:**
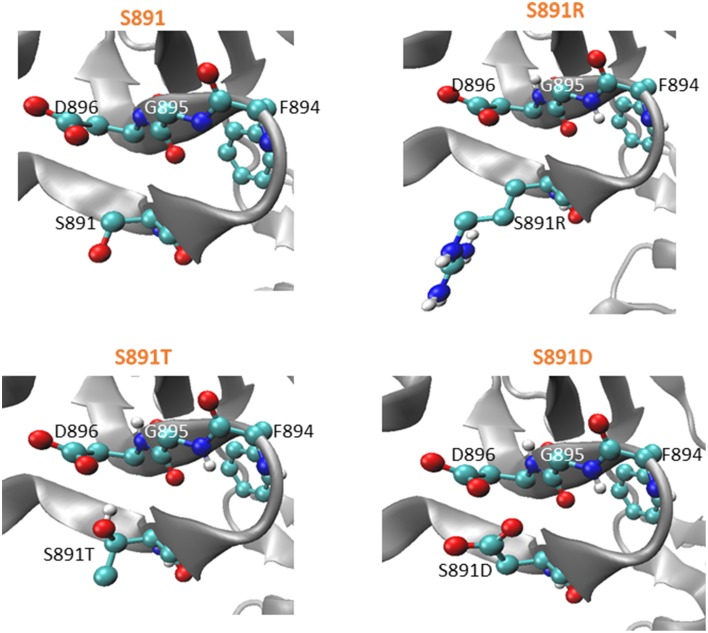
**Modeled structure of the G-loop of BRI1 in the ATP-bound form with various amino acids at the 891 position**. SCWRL4 was used to predict side chain conformations in the structure determined for the T872A mutant of BRI1 (4OAB) (Bojar et al., 2014). Zoomed-in views of spatial orientations of 891S or mutated residues in the mutant models as indicated. Coloring scheme: red, oxygen; white, carbon; blue, nitrogen.

Using the coordinates of the native BRI1 crystal structure (Bojar et al., 2014), the impact of the S891T directed mutation was modeled using SCWRL4, which predicts amino acid side chain conformations based on electron density and free energy considerations (Krivov et al., 2009). In the ATP-bound form, the Ser side chain oxygen is pointing downward, whereas the side chain oxygen of the Thr side chain (in the S891T mutant) is predicted to be pointing upwards (Figure 5, left panel). It is possible that this difference in side chain orientation impacts kinase activation kinetics even when the residues are not themselves phosphorylated. In addition, once phosphorylated it is possible that the upward-pointing phosphothreonine would interfere more strongly with the negatively-charged side chain of Asp-896 that is located above it. The structural modeling also suggests that the basic side chain in the S891R mutant is pointing downward, whereas the acidic side chain of the S891D mutant is pointing upward (Figure 5, right panel). The proximity of the acidic side chains of the two Asp residues in the S891D mutant would be consistent with the inhibition of kinase activity observed (Oh et al., 2012c).

### Expression of BRI1 (S891T)-Flag partially rescues *BRI1-5*

**Figure 6:**
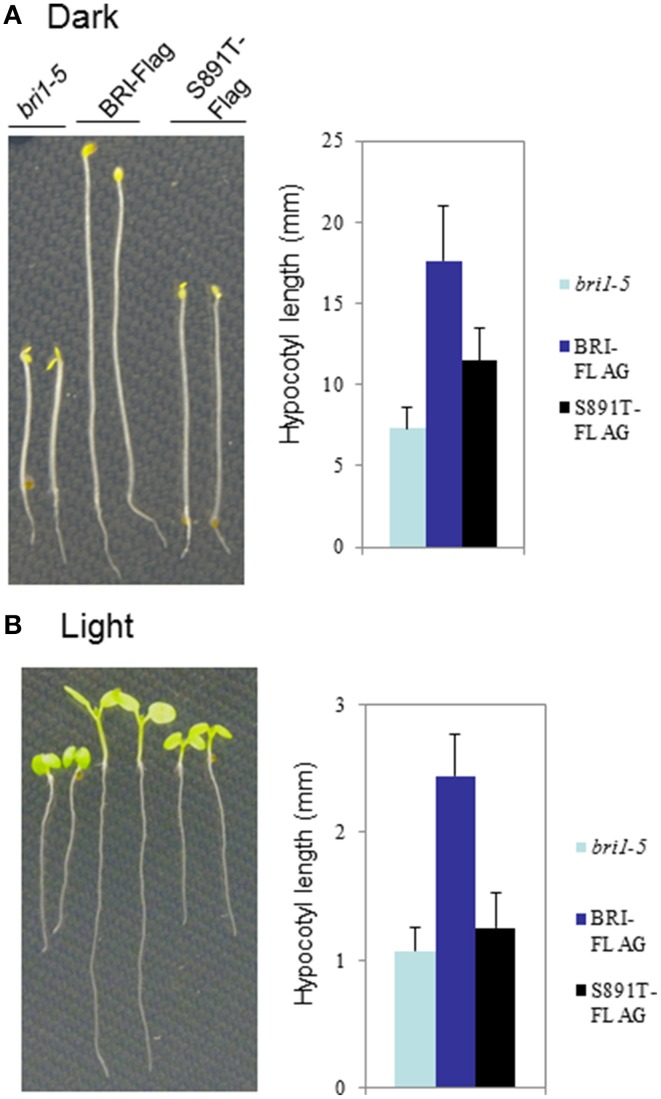
**Altered hypocotyl growth in BRI1 (S891T)-Flag plants**. Seedlings of *bri1-5*, wild type BRI1-Flag or BRI1 (S891T)-Flag were grown for 7 days in the **(A)** dark or **(B)** light (16 h photoperiod). Seedlings were grown on vertical plates containing 1.2% agar with half-strength MS media.

**Figure 7:**
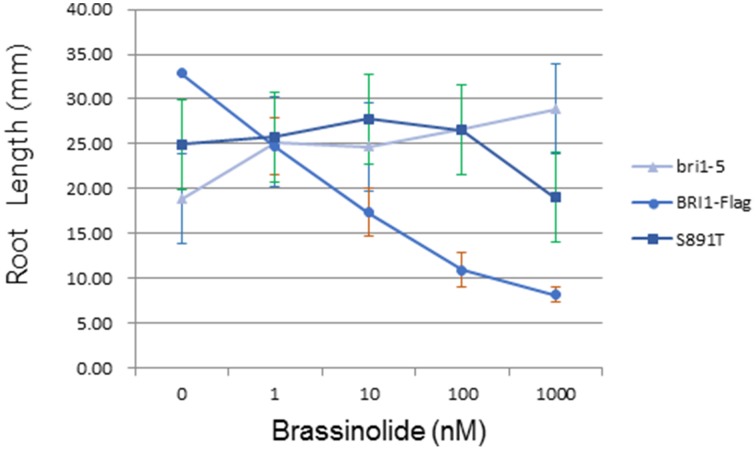
**Inhibition of root elongation by high concentrations of BL**. Root elongation in *bri1-5* plants was insensitive to BL as a result of impaired BR signaling. Transgenic plants expressing Flag-BRI1 (S891T) were substantially less sensitive to BL compared to plants expressing wild type Flag-BRI1.

The *bri1-5* mutant (Noguchi et al., 1999) is impaired in BR signaling as a result of ER-retention and degradation of the BRI1(C69Y) protein (Hong et al., 2008). As a result, *bri1-5* seedlings and plants have characteristic BR-signaling phenotypes; seedlings grown in the dark have short hypocotyls with open cotyledons and plants grown in soil are dwarfs with short petioles and round leaves (Clouse, 2011). These characteristic growth phenotypes can be rescued by transformation of *bri1-5* plants with wild-type BRI1-Flag (Wang et al., 2005a,b, 2008). Transformation of *bri1-5* plants with BRI1 (S891T)-Flag partially rescued hypocotyl growth in seedlings grown in the dark (Figure 6A) or light (Figure 6B). However, dark-grown seedlings of the S891T plants had open cotyledons similar to the *bri1-5* plants, and in contrast to the BRI1-Flag plants. As another physiological read out of BR signaling we examined the inhibition of root elongation by high concentrations of BL (Clouse et al., 1996; Li et al., 2001). As shown in Figure 7 concentrations of BL above 1 nM inhibited root elongation in the wild type BRI1-Flag plants with 50% inhibition of root elongation achieved at about 10 nM BL. In contrast, root elongation in the *bri1-5* plants was insensitive to BL over the range tested, and S891T plants showed partial inhibition only at the highest concentration of BL tested (1000 nM). Collectively, these results suggest that BR signaling was impaired in plants expressing BRI1 (S891T)-Flag in the *bri1-5* background.

**Figure 8:**
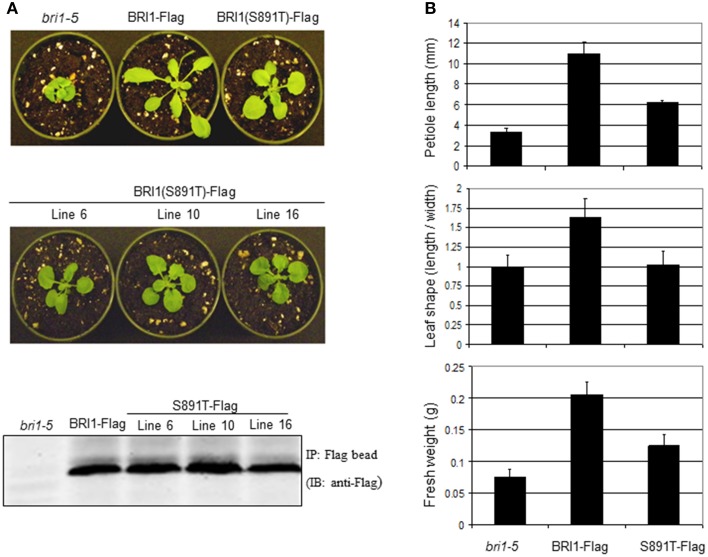
**Expression of BRI1 (S891T)-Flag affects rosette growth of vegetative Arabidopsis plants. (A)** Vegetative morphology of *bri1-5*, BRI1-Flag, and several independent lines of BRI1 (S891T)-Flag plants after 21 days of growth with a long day photoperiod (16 h light/8 h dark). To confirm protein level of BRI1-Flag and BRI1 (S891T)-Flag, *A. thaliana* plants were grown in shaking liquid culture and microsomal membranes were isolated. Membranes were solubilized with 1% (vol/vol) Triton X-100, clarified by centrifugation. BRI1-Flag and BRI1 (S891T)-Flag were then immunoprecipitated with prewashed anti-Flag M_2_ affinity gel (Sigma–Aldrich). Immunoprecipitated full-length BRI1-Flag and BRI1 (S891T)-Flag proteins were subjected to SDS-PAGE followed by transfer to PVDF membranes and immunoblot analysis performed using anti-Flag antibodies (1:5000 dilution), **(B)** Morphometric analysis of vegetative plants similar to those shown in **(A)**.

Rosette size and morphology of S891T plants growing in soil were also suggestive of impaired BR signaling. Several independent transgenic lines expressing BRI1 (S891T)-Flag in the *bri1-5* background were found to be intermediate between the *bri1-5* mutant and plants expressing wild-type BRI1-Flag (in the *bri1-5* background; Figures 8A,B). The partial rescue by expression of BRI1 (S891T)-Flag was apparent in terms of rosette fresh weight and petiole length whereas there was no restoration of the wild type leaf shape (length/width ratio).

### Normal expression of BRI1 (S891T)-Flag transcript and protein

**Figure 9:**
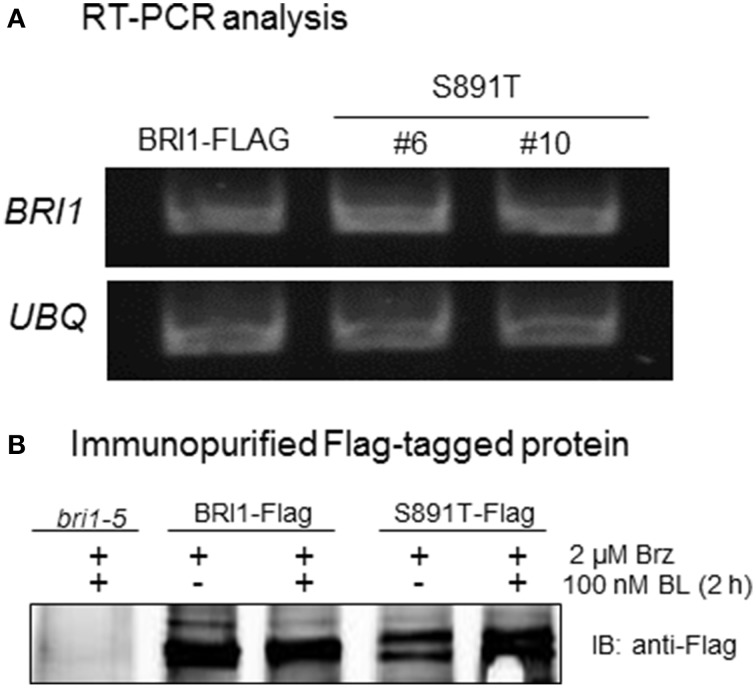
**Analysis of BRI1 transcript and protein levels in transgenic plants. (A)** RT-PCR analysis of BRI1 mRNA steady state expression level in BRI1-Flag and two lines of BRI1 (S891T)-Flag plants harvested 7 days after germination. **(B)** Anti-Flag immunoblots of wild type BRI1 and the S891T mutant protein isolated from microsomal membranes by affinity purification. Seedlings were grown in liquid culture for 7 days, treated with brassinazole (Brz) for 4 days, and harvested before and 2 h after addition of 100 nM BL.

Analysis of leaf tissue by RT-PCR established that the steady state transcript levels of BRI1-Flag and the S891T mutant were similar (Figure 9A). Likewise, expression levels of the wild type BRI1-Flag and BRI1 (S891T)-Flag protein in liquid culture seedlings were generally similar to one another both in the absence of endogenous BL and 2 h after addition of exogenous BL (Figure 9B). Thus, partially impaired BR signaling in the S891T plants cannot be readily attributed to large alterations in levels of the receptor kinase. The possibility that subtle changes in receptor kinase levels that may be difficult to discern with the sensitivity of our assay methods could contribute to the lack of full complementation by BRI1 (S891T) seems unlikely given recent results (Bücherl et al., 2013) suggesting that only a small fraction of the total BRI1 is engaged in signaling.

## Concluding remarks

The function of phosphosites is usually tested by substituting a conservative but non-phosphorylatable residue at the site or an acidic residue that may function as a phosphomimetic. This has been applied to many phosphosites in receptor kinases, including BRI1 and BAK1. A more subtle modification is to substitute a Thr for a Ser (and vice versa) as was done in the present study. To our knowledge this is the first time this type of substitution has been tested with a plant receptor kinase. While one might suspect that such a subtle change would have no functional consequences, we were surprised to find significant impact on BRI1 activity. The two key observations to emerge are: (1) the recombinant Flag-BRI1 (S891T) protein autophosphorylated/activated more slowly than wild type Flag-BRI1 but ultimately attained the same activity level; and (2) when stably expressed in transgenic *bri1-5* plants, the BR-signaling phenotype was only partially rescued supporting the idea that the S891T mutant is not functionally equivalent to the wild type BRI1 (S891) protein. Given that the G-loop is strictly conserved across BRI1 orthologs in higher plants (Supplementary Figure 1), it seems likely that phosphorylation of the G-loop Ser might be a conserved regulatory mechanism and thus the results of the present study outline a possible new avenue for engineering BR signaling in crop species. Our working model is that the slower rate of activation of S891T results in impaired BR signaling. However, whether this results from a direct effect of the unphosphorylated Thr side chain, which is predicted to orient differently from the Ser side chain (Figure 5), or whether it is related to phosphorylation of the Thr-891 residue remains to be determined. Although beyond the scope of the present study, we have the tools and platform to test these possibilities. In general terms, our results also establish that Ser/Thr substitutions are worth exploring as the residues may not be functionally similar for all phosphosites and may provide for an interesting strategy to engineer receptor kinase function.

### Conflict of interest statement

The authors declare that the research was conducted in the absence of any commercial or financial relationships that could be construed as a potential conflict of interest.

## Supplementary material

Figure S1 **Conservation of the G-loop sequence including Ser-891 of Arabidopsis BRI1 across BRI1 orthologs**.
