# Supplementary figures and images for: Functional analysis of the BRI1 receptor kinase by Thr-for-Ser substitution in a regulatory autophosphorylation site
Source: Front Plant Sci. 2015 Jul 30;6:562. doi: 10.3389/fpls.2015.00562 (PMC4519688; doi:10.3389/fpls.2015.00562)

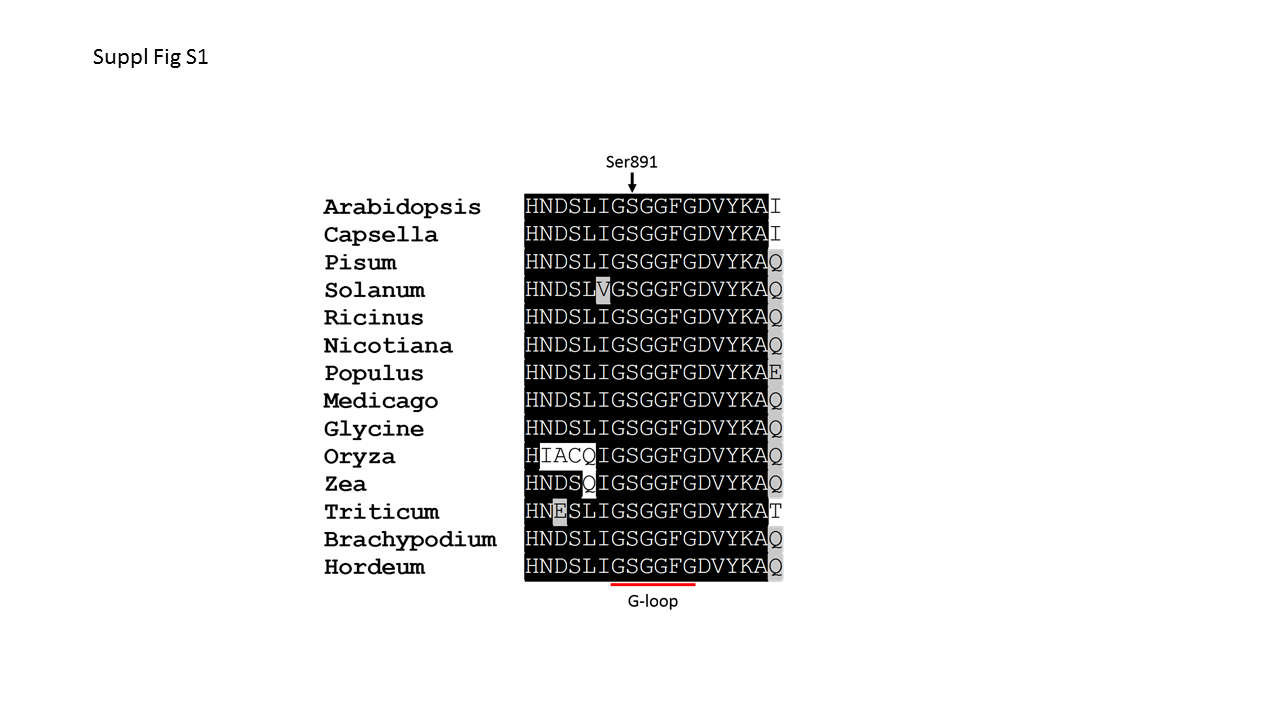

Supplement: Figure S1 — Conservation of the G-loop sequence including Ser-891 of Arabidopsis BRI1 across BRI1 orthologs. [file Image1.TIF]
